# Joint cartilage thickness and automated determination of bone age and bone health in juvenile idiopathic arthritis

**DOI:** 10.1186/s12969-017-0194-9

**Published:** 2017-08-10

**Authors:** Marinka Twilt, Dan Pradsgaard, Anne Helene Spannow, Arne Horlyck, Carsten Heuck, Troels Herlin

**Affiliations:** 1 Department of Paediatrics, Section of Rheumatology, Alberta Children’s Hospital, University of Calgary, Calgary, AB Canada; 2 Department of Paediatrics, Division of Rheumatology, Aarhus University Hospital, Aarhus, Denmark; 3 Department of Radiology, Aarhus University Hospital, Aarhus, Denmark; 4 Pediatric Rheumatology Clinic, Department of Pediatrics, Aarhus University Hospital, Palle Juul-Jensens Boulevard 99, DK-8200 Århus N, Denmark

**Keywords:** Juvenile idiopathic arthritis, Joint cartilage, Bone age, Bone health index, Musculoskeletal ultrasonography

## Abstract

**Background:**

BoneXpert is an automated method to calculate bone maturation and bone health index (BHI) in children with juvenile idiopathic arthritis (JIA). Cartilage thickness can also be seen as an indicator for bone health and arthritis damage. The objective of this study was to evaluate the relation between cartilage thickness, bone maturation and bone health in patients with JIA.

**Methods:**

Patients with JIA diagnosed according ILAR criteria included in a previous ultrasonography (US) study were eligible if hand radiographs were taken at the same time as the US examination. Of the 95 patients 67 met the inclusion criteria.

**Results:**

Decreased cartilage thickness was seen in 27% of the examined joints. Decreased BHI was seen in half of the JIA patient, and delayed bone maturation was seen in 33% of patients. A combination of decreased BHI and bone age was seen in 1 out of 5 JIA patients. Decreased cartilage thickness in the knee, wrist and MCP joint was negatively correlated with delayed bone maturation but not with bone health index.

**Conclusion:**

Delayed bone maturation and decreased BHI were not related to a thinner cartilage, but a thicker cartilage. No relation with JADAS 10 was found. The rheumatologist should remain aware of delayed bone maturation and BHI in JIA patients with cartilage changes, even in the biologic era.

## Background

Juvenile Idiopathic Arthritis (JIA) is the most common rheumatic disease in childhood. It can result in musculoskeletal pain, joint stiffness and swelling and decreased range of motion. If left untreated JIA will lead to disability of the affected joints. If kept untreated, chronically inflamed synovium and involvement of associated muscle and soft tissues, eventually leading to degeneration of the osteocartilaginous structures, are the primary cause for functional disability in JIA [1]. In pediatric rheumatology it is of great importance to follow osteocartilaginous degeneration [2]. The imaging modalities frequently used to visualize this process are conventional radiography, magnetic resonance imaging (MRI), and within the last decade ultrasonography (US). US is of particular benefit in the assessment of early signs of arthritis, such as joint effusion and synovial thickening [3]. Unlike with the MRI, without using any contrast, hyperemia in the synovial microcirculation as part of the inflammatory process can be detected using the color Doppler or the power Doppler technique [4]. US is relatively inexpensive and does not require any radiation. US is well tolerated, even in the very young patients, can be used to view different joints in one session, and can be viewed at the bedside or in the clinic room.

High frequency US can be used to easily view the joint cartilage as an anechoic structure, as cartilage has a high water content. In previous studies, we validated the assessment of cartilage thickness by US in a pediatric setting [5, 6]. We found a low intra- and inter-observer variability and a good level of agreement, with no significant systematic joint size-related differences in cartilage thickness measurements between MRI and US [7, 8]. Based on a large cohort of healthy children we established age- and sex-related normal reference values for cartilage thickness of the knee, ankle, wrist, second metacarpophalangeal (MCP) and second proximal interphalangeal (PIP) joints [5]. In a subsequent study we measured cartilage thickness of those joints in JIA patients and found reduced cartilage thickness in children with JIA compared to healthy controls [2, 8]. Children with polyarticular or systemic JIA had thinner cartilage than children with oligoarticular JIA [2].

Continuous exposure to inflammatory cytokines, together with glucocorticoid therapy, affects bone formation [9–11]. This, combined with decreased physical activity and pubertal delay puts JIA patients at increased risk of impaired growth and reduced bone mineral density (BMD) [12]. The assessment of bone age is usually made using the Greulich and Pyle atlas [13]. Dual-energy X-ray densitometry (DXA) is the most commonly used method to assess BMD [14]. Recently, BoneXpert was developed bringing back the use of radiogrammetry, one of the oldest methods to assess BMD. This new digital X-ray radiogrammetry (DXR) method combines the assessment of bone-age with radiogrammetric assessment of (cortical) BMD) of the second to the fourth metacarpal joints [15, 16]. BoneXpert expresses the cortical BMD as a Bone Health Index (BHI), in which cortical BMD is corrected for size. The BoneXpert method makes use of conventional radiography of the hand, thereby making it attractive due to relatively low costs, and lower effective radiation dose compared to other effective methods [16]. The application has been tested in healthy pediatric populations and in the JIA population and showed to be a reliable method for assessing bone age and cortical BMD [17–19]. BoneXpert method is an easy-to-use method provided that radiographs are of reasonable quality and the patients’ bone age lies within the age ranges of the program [17, 18]. The JIA population, derived from a biologic registry (Dutch) ABC register showed a delayed bone maturation and lower cortical BMD than healthy patients [19].

In the literature no reports are recorded to look at the relationship between cartilage thickness and bone health in JIA.

Therefore, the aim of our study was to evaluate bone maturation and bone density by using BoneXpert in a cross-sectional JIA population and correlate these findings to the cartilage thickness assessed by US.

## Methods

### Patients

Children diagnosed with JIA according to the 2001 revised International League of Associations for Rheumatology (ILAR) classification aged 5–15 years and followed at the pediatric rheumatology department of Aarhus University Hospital, Denmark, were invited to participate [20]. Inclusion criteria were; systemic JIA, persistent and extended oligoarticular JIA, and rheumatoid factor (RF)- positive or RF-negative polyarticular JIA. Informed consent was received from the parents. Patients were excluded if they had received an intra articular corticosteroid injection (IACI) within 1 month prior to examination or had a history of previous joint surgery.

Demographic information was retrieved from the medical records and included; disease subtype, age at onset, disease duration. The history of affected joints during the disease course was recorded.

Joint activity was assessed by an experienced pediatric rheumatologist on the same day as the US and BoneXpert x-ray. Joint activity was defined as swelling within a joint, or limitation in the range of joint movement with joint pain or tenderness. The Juvenile Arthritis Diseases Activity Score (JADAS) was established for every patient, consisting of the active joint count of the 10 joints that were scanned by US, ESR, parents’ global assessment, and physician’s global assessment.

### US examination

A Hitachi EUB 7500 scanner with a 6–14 MHz linear transducer (EUP-L65) was used for the US measurements. All US examinations were performed by the same observer throughout the study (DOP). The patients were examined as described, using the same US settings (European League Against Rheumatism (EULAR) standards) described in a healthy age- and sex-matched cohort [5–7]. This ensured consensus with respect to cartilage thickness measurements. The US scans were performed with the observer blinded to the clinical information regarding JIA subtype, joint status, disease duration, and treatment. The images were saved and stored anonymously on a hard drive using 9-digit code. The pressure of the probe was adjusted to a level just below visible deformation on the anatomical structure. Greyscale examinations of distal femoral cartilage (knee joint), anterior talar cartilage (ankle joint), proximal dorsal scaphoid bone cartilage (wrist joint), distal second metacarpal cartilage (second MCP joint), and distal cartilage of the second proximal interphalangeal bone (second PIP joint). Cartilage thickness was measured in mm. Standard scans of hyaline cartilage thickness are based on the guideline recommendations by EULAR [21].

### BoneXpert

The stand-alone Windows product of BoneXpert (BoneXpert Version 2.1.0.12;Visiana, Holte, Denmark) was used to analyze the hand radiographs. BoneXpert automatically generates the following outcome variables: (calendar) age, bone age based on Tanner and Whitehouse (TW) method [22] and Greulich and Pyle (GP) method [13], Z-scores of GP bone age (compared with a healthy reference population: girls <15 years, boys <17 years) [23], BHI and Z-score of BHI [15]. BHI is based on cortical thickness (T) of the three middle metacarpal bones. To compensate for the high variation in stature of growing children BoneXpert incorporated the metacarpal width (W) and length (L) in the construction of BHI [16]:

$$ \mathrm{BHI}=\uppi \mathrm{T}\left(1-\mathrm{T}/\mathrm{W}\right)/{\left(\mathrm{LW}\right)}^{0.33} $$

**Fig. 1:**
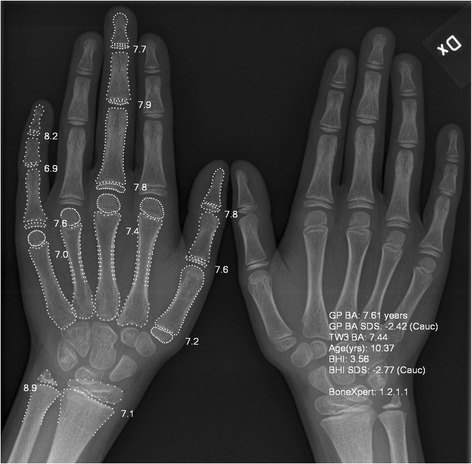
Digital X-ray radiogrammetry of the left and right hand of a patient with JIA. BoneXpert® software was used to perform automated determination of bone age based on the radius, ulna and the bones in ray 1, 3 and 5 (marked with *white dots*). Bone health index (BHI) is based on the measurements of cortical thickness in the three middle metacarpals

The complete hand and wrist joint of both the left and right sides were included in the radiographs (Fig. 1).

### Statistics

Descriptive statistics are reported in terms of absolute numbers, median and interquartile range (IQR). To determine whether the Z-score of cartilage thickness or bone age and BHI were different from those in the healthy population, a one sample t-test was used. Correlation coefficient (Spearman’s Rho) was used to determine the correlation between Z-score of cartilage thickness and bone age and BHI. IBM SPSS Statistics, version 24 (IBM Corp, Armonk, NY, USA) was used for all analyses.

## Results

This study was performed within a larger study on the use of US in JIA. In this study 155 patients with JIA aged between 5 and 15 years were invited to participate, 52 patients declined, 4 had unclassified arthritis, and 5 canceled their scheduled appointment due to illness. As a result, 94 patients were included in the initial study. In 27 patients digital radiogrammetry of the hand was not performed. Therefore, a total of 67 patients were evaluated by US and BoneXpert and included in the current study. The study included 16 boys and 51 girls, with a median age at investigation of 10.9 years (range 7.8–14.4 years), and a median disease duration of 41 months. Patient demographics, including distribution of JIA subcategories, are described in Table 1.

**Table 1:** Demographic data for study patients (*n* = 67)

	Number	Age (months)	Disease duration (months)
All	67 (16 boys, 51 girls)	131 (94–168)	41 (15–80)
Oligo-persistent	25	107 (92–144)	15.5 (6.5–47.5)
Oligo-extended	13	157 (118.5–176)	83 (54.5–133.5)
Polyarticular RF-neg	17	128 (120–173.5)	51 (23.5–87)
Polyarticular RF-pos	4	167 (149–183)	20.5 (7–26.5)
Systemic	8	107.5 (85–172.5)	48 (29–117.5)

Values are expressed as median (interquartile range)

A total of 680 joints were assessed by US for cartilage thickness. Decreased cartilage thickness compared to healthy age-matched controls [5–7] < −1 SD) was present in 27% of the examined joints (181 joints). The most common joint with decreased cartilage thickness was the second PIP joint, followed by the wrist. Decreased cartilage thickness was uncommon in the ankle joint (Table 2). If controlling for age and gender a significant decreased cartilage thickness was seen in the knees, wrists and second PIP joints, but not in the ankles or second MCP joints (Table 2). We found a highly significant correlation between the cartilage thickness measured in either the right- or left knee, right or left ankle, right or left wrist and right or left finger joints (*r* = 0.561–0.884, *p* < 0.001).

**Table 2:** US measurement of joint cartilage thickness in children with JIA

Joint	Median (IQR)	*t*	*p*
Knee R	- 1.927 (− 2.50 – (− 1.06))	- 10.465	< 0.001
Knee L	- 1.63 (−2.31 – (−0.71))	- 9.203	< 0.001
Ankle R	0.13 (− 0.7–1.25)	0.933	0.458
Ankle L	0.20 (− 0.96 – (1.19))	1.234	0.325
Wrist R	- 0.81 (− 1.45 – (− 0.10))	- 6.513	< 0.001
Wrist L	- 0.89 (− 1.63 – (− 0.22))	- 8.277	< 0.001
MCP R	0.02 (− 1.01–1.15)	0.508	0.705
MCP L	0.02 (− 0.89–1.16)	1.482	0.142
PIP R	- 2.24 (− 3.27 – (− 1.39))	- 13.254	< 0.001
PIP L	- 2.19 (− 3.50 – (−1.27))	- 11.969	< 0.001

One sample t-test. US assessed joint cartilage thickness when controlled for age and gender expressed as median Z-score (interquartile range). *N* = 67

The automated bone age, using either the Greulich-Pyle or Tanner-Whitehouse method, correlated closely (*r* = 0.992, *p* < 0.001) and showed significantly lower bone age in JIA patients compared to healthy controls, when controlled for age and gender (Table 3). Delayed bone maturation was found in 22 (33%) patients (bone age < −1SD) of which 10 patients had a bone age < −2SD. BHI was decreased in children with JIA (Table 3). Thirty-four (50.7%) of the patients showed a decreased BHI below −1 SD of which 9/66 had a BHI < −2SD. Fourteen patients demonstrated a combination of a decreased BHI and a decreased bone age.

**Table 3:** Automated bone age and bone health index as assessed by digital radiogrammetry. *N* = 67

		Median (IQR)	*t*	*p*
Age (years)	67	10.9 (7.8–14.4)		
Bone age GP	67	9.98 (7.28–13.86)		
Bone age GP (SDS)	63	- 0.49 (− 1.32–0.25)	- 2.875	0.006
Bone age TW	67	9.65 (7.03–13.5)		
Bone health Index	66	4.35 (4.06–4.70)		
Bone health Index (SDS)	66	- 1.02 (− 1.60 – (− 0.30)	- 7.199	< 0.001
Disease duration (months)	67	41 (15–80)		

*GP* Automated digital Greulich-Pyle bone age method. *TW* Automated digital Tanner-Whitehouse bone age method. *SDS* standard deviation score. SDS for the Tanner Whitehouse bone age method is not available. One sample t-test

The decreased bone age was significantly correlated with a longer disease duration. This was not seen for the BHI. Both the bone age and BHI were not correlated to JADAS 10 (*r* = 0.056, *p* = 0.67 and *r* = 0.082, *p* = 0.52, respectively.

Decreased cartilage thickness measured by US in the knee, wrist and second MCPs was significantly negatively correlated with standardized bone age but this was not found for the ankle and 2nd PIP joints (Table 4). There is no difference in cartilage thickness and BHI, regardless whether BHI was < − 1 SD or < − 2 SD. Thus, no correlation was observed between cartilage thickness and BHI (table 4). Children with a bone age < −1 SD (*n* = 22) showed a significantly increased cartilage thickness in the right knee, both wrists, and both second MCPs, compared to children with a bone age > −1 SD (not shown). Decreased bone health and bone age were more pronounced in Tanner stage 1–3 (Table 5).

**Table 4:** Correlation between US-assessed joint cartilage thicknesses measured as Z-score and Bone age and bone health index assessed by digital radiogrammetry. *N* = 67

US mean Z score	Bone age GP SDS		BHI SDS	
	r	*p*	r	*p*
Knee	- 0.288	0.023*	- 0.069	0.587
Ankle	- 0.172	0.177	- 0.222	0.073
Wrist	- 0.298	0.018*	- 0.121	0.331
2nd MCP	- 0.500	<0.001*	0.019	0.880
2nd PIP	- 0.058	0.649	−0.009	0.945

US mean Z score: mean of the Z score of joint cartilage thickness assessed by ultrasonography of the left and right joint. *GP* Greulich-Pyle, *BHI* Bone health index, *SDS* standard deviation score. *: significant correlation *p* < 0.05

**Table 5:** Tanner stage and automated bone age and bone health index determined by digital radiogrammetry in juvenile idiopathic arthritis

Tanner stage	Number	Bone age GP	Bone age GP (SDS)	BHI SDS	BMI
1	33	7.3 (5.8–8.2)	−0.83 (−1.57-(−0.08))	−0.75 (−1.57-(−0.33))	15.9 (14.8–16.4)
2	12	11.0 (10–11.7)	−0.41 (−1.1–0.17)	−1.28 (−2.13-(−0.83))	17.8 (16.1–20.2)
3	7	13.9 (12.1–14.9)	−0.88 (−1.36-(−0.28))	−1.48 (−1.58–0.30)	19.2 (16.9–19.7)
4	9	15.5 (13.8–16.7)	0.26 (−0.76–1.12)	0.94 (−1.52–0.05)	18.4 (16.2–19.3)
5	6	16.8 (16–18)	2.33 (1.08–2.54)	−1.47 (−2.15–0.28)	25.1 (20.4–29.5)

*GP* Greulich-Pyle, *BHI* Bone health index, *SDS* standard deviation score, *BMI* body mass index

## Discussion

This is the first paper to investigate the relation between cartilage thickness measured by US and automated bone maturation and bone health index. JIA patients show a thinner cartilage than healthy controls. One in two patients showed a decreased BHI (<= −1SD), indicating the heavy burden of JIA on the bone health of these patients. Notably they have a lower cartilage thickness in their second MCP joint, this is one of the MCP joints that are used to to calculate the BHI. Interestingly, there is no correlation between cartilage thickness and BHI. This might be explained by the 3rd and 4th metacarpal joint, which are taken into account while calculating the BHI, but were not assessed by US.

Previous studies have shown that patients with JIA are known to have a delayed bone maturation [9–12]. This was, however, not studied in relation to the cartilage thickness. Surprisingly, in the knee, wrist and MCP there is a significant correlation between a thicker cartilage and a delayed bone maturation, which is in contrast with one would have expected. However, in JIA, enhanced focal bone maturation is a well-known phenomenon as a result of joint inflammation, which may lead to accelerated thinning of the cartilage. One in five JIA patients showed both a decreased bone age and a decreased BHI.

The BoneXpert method used to measure bone maturation and BHI is an easy to use, high precision method. This study is one of the first research studies to demonstrate the use of the BoneXpert method in JIA. Nusman et al. reported on the feasibility of this method in JIA and the small difference between left- and right-hand radiographs [19].

The ongoing delayed bone maturation and decreased BHI in JIA patients with relatively mild disease, and no correlation with JADAS 10 score, shows the need for ongoing bone health strategies in JIA. Early on, corticosteroids were blamed for the bone health issues in JIA, however with the new treatments available and the continuing decreased usage of corticosteroids, bone health in JIA is still under threat.

This study is limited by the very specific population studied within an US cohort as previously described [2, 8]. These factors introduce a selection bias, which limits the generalizability to the full JIA population. However, patients included in this cohort were recruited consecutively in clinic and the subgroup evaluated in this study is a representation of that group. This group, however is a group of patients with longer standing diseases and may not represent the new JIA patient. Another limitation is that degradation of the cartilage may not appear uniformly. The degradation might affect the joint more severely on either side of the scanned area. Unlike the MRI, US cannot visualize the entire cartilage surface, which was a limitation of the study.

## Conclusion

This first study on cartilage thickness, bone maturation and bone health shows the ongoing delayed bone maturation and decreased BHI in JIA patients. The delayed bone age is not related to a thinner cartilage, but a thicker cartilage assessed by US. Rheumatologist need to be aware of the ongoing issue with bone health in JIA patients, even in the biologic era.

## Abbreviations

BA: Bone age
BHI: Bone health index
BMD: Bone mineral density
DXA: Dual energy X-ray densitometry
DXR: Digital X-ray radiogrammetry
ESR: Erythrocyte sedimentation rate
EULAR: European League Against Rheumatism
GP: Greulich-Pyle
IACI: Intraarticular corticosteroid injection
ILAR: International League of Associations for Rheumatology
IQR: Interquartile range
JADAS: Juvenile arthritis disease activity score
JIA: Juvenile idiopathic arthritis
MCP: Metacarpophalangeal joint
MRI: Magnetic resonance imaging
PIP: Proximal interphalangeal joint
RF: Rheumatoid factor
SD: Standard deviation
TW: Tanner-Whitehouse
US: Ultrasonography
